# Development and validation of a fast quantitative real-time PCR assay for the detection of African swine fever virus

**DOI:** 10.3389/fvets.2022.1037728

**Published:** 2023-01-04

**Authors:** Hyun Jin Hwang, Yun Seong Choi, Kyungyoung Song, Maciej Frant, Jeong Hee Kim

**Affiliations:** ^1^R&D Center, Ahram Biosystems Inc., Seoul, South Korea; ^2^Department of Swine Diseases, National Veterinary Research Institute, Puławy, Poland; ^3^Department of Oral Biochemistry and Molecular Biology, School of Dentistry, Kyung Hee University, Seoul, South Korea; ^4^Department of KHU-KIST Converging Science and Technology, Graduate School, Kyung Hee University, Seoul, South Korea

**Keywords:** African swine fever virus, real-time PCR, fast, quantitative, diagnostics

## Abstract

African swine fever virus (ASFV) is a double-stranded DNA virus that causes African swine fever (ASF), a lethal hemorrhagic fever that is highly contagious among domestic pigs and wild boars. Due to the high mortality rates and highly contagious nature of the ASF, it is important to develop a fast detection method for ASFV with high sensitivity and specificity to take an immediate action to stop wide spread of the virulent disease. Therefore, a fast and quantitative molecular detection method of ASFV is presented in this study. A total of 24 genotypes of ASFV have been identified based on nucleic acid sequences of the major capsid protein p72. The primers and probe of the present assay was designed to detect all of the p72-based genotypes of ASFV. The turnaround time for PCR detection was within 50 min which is at least about two-times faster compared to other PCR assays. Limit of detection (LoD) was 6.91 genomic copies/reaction for the most virulent genotype II. LoD values for other genotypes were within 10–20 copies/reaction. Cross-reactivity of the assay was validated using a panel of pathogens related to swine disease, and no cross-reactivity was observed. Positive and negative clinical samples (50 samples each) obtained from sick and healthy animals, were used to validate the assay. The results showed that 100% agreement for both positive and negative samples. In summary, the assay described in this study offers the advantage of rapid detection of all genotypes of ASFV with high sensitivity and specificity. The assay is a valuable tool both in clinical and laboratory uses for sensitive and fast detection of ASFV.

## Introduction

African swine fever (ASF) is a highly contagious viral disease caused by ASF virus (ASFV), an enveloped virus with a large, double-stranded DNA which belongs to the *Asfarviridae* family (1, 2). The clinical symptoms include high fever, anorexia, vomiting, diarrhea, and hemorrhage. These clinical symptoms and post-mortem findings are difficult to distinguish from those of classical swine fever (CSF). ASFV is highly contagious and causes high mortality rates: close to 100% in domestic pigs and approximately 95% in wild bores. Therefore, it is under strict surveillance under Office International des Épizooties (OIE, World Organization of Animal Health). ASF is a serious threat for pork production industry and causes a significant economic impact globally (2–4).

Total of 24 distinct genotypes of ASFV have been identified based on sequences of the gene encoding the major capsid protein p72. Eight serotypes are also known based on viral hemagglutinin CD2-like protein (CD2v) and C-type lectin (2, 5, 6). Among these, genotype II stain is highly virulent and is prevalent in Europe, Russia, China, and Southeast Asia (7–12).

Currently, no vaccine or treatment is available for ASF. Therefore, development of fast and reliable molecular diagnostics method is critical to timely apply the control measures and to prevent wide spread among domestic and wild pigs. In addition, since there are similarities in clinical symptoms between ASF and other swine diseases, it is important to have rapid and specific diagnosis for timely implementation of follow-up measures.

The traditional methods for diagnosis of viral diseases were generally based on virus isolation. However, this process is labor-intensive and time-consuming (13, 14). And not all viruses can be isolated from the samples. Conventional Polymerase chain reaction (PCR)-based technology have been used for the detection of infectious diseases including ASF. PCR technology can be applicable for samples that are not suitable for virus isolation. However, detection of ASFV with conventional PCR involves extra post-PCR steps such as electrophoresis, and the sensitivity was generally much lower than the real-time PCR method (15–17).

An alternative technology for ASFV detection is loop-mediated isothermal amplification (LAMP) (18, 19). Recently, application of CRSPR-Cas12a coupled with LAMP was shown to give an enhanced fluorescence assay sensitivity (20, 21). Recombinase polymerase amplification (RPA) combined with a lateral flow strip for ASF detection was also introduced (22, 23). However, the drawback of these methods is that they require a larger number of primers with more complicated primer design. Moreover, LAMP and RPA showed lower sensitivity compared to PCR assays (24). Real-time PCR technology has been most widely used for the detection of infectious diseases including ASFV because of its high sensitivity and specificity (14, 25–29). However, the reported studies of real-time PCR detection of ASFV involved relatively long reaction times, approximately from 1 and half hours to 2 h (14, 25, 29).

In this study, we developed and validated a fast and quantitative real-time PCR test that can detect all currently known 24 genotypes of ASFV utilizing a new chemistry including a fast Taq polymerase. ASFV detection can be completed within 50 min without losing its sensitivity and specificity.

## Materials and methods

### Synthetic double stranded DNA template

Synthetic double-stranded DNAs for different genotypes of ASFV p72 gene were used for analytical performance tests of the assay. Twenty-four genotypes of ASFV p72 gene were classified into seven genotype groups based on nucleic acid sequences of the p72 target region (Table 1). The seven genotype groups were designated as GT2, GT3, GT7, GT8, GT9, GT10 and GT23, referring to the representative genotype of each group. A double-stranded DNA of the p72 conservative region with a size of about 400 bp (gBlocks, IDT, Singapore) was synthesized for each genotype group and used as a standard sample. The synthesized DNAs were dissolved in nuclease-free water (Molecular Biology grade, Sigma-Aldrich, USA) to the concentration of 1 × 10^9^ copies/μL, aliquoted and stored at −70°C.

**Table 1:** Classification of 24 genotypes of the genes for ASFV p72 protein.

**Group name**	**Representative p72 genotype**	**p72 genotypes**
GT2	II	I, II, XVII, XVIII
GT3	III	III, IV, V, VI, XIX, XX, XXI, XXII, XXIV
GT7	VII	VII
GT8	VIII	VIII, XI, XII, XIII, XIV, XV, XVI
GT9	IX	IX
GT10	X	X
GT23	XXIII	XXIII

### Primer and probe design

The sequences of the seven known groups of ASFV within the conserved region of ASFV major capsid protein p72 were searched. Primers and TaqMan probe were designed to target the conserved region and also to match all 7 genotype groups of ASFV listed in Table 1. The sequences of forward and reverse primers and probes (from LGC Biosearch Technologies, USA) are listed in Table 2. A 1:1 mixture of two forward primers, ASFV-F1 and ASFV-F2, were used for PCR. For internal PCR control (IPC), primers and probe were designed to target human tubulin alpha 1a gene (NCBI Reference Sequence: NG_008966.1).

**Table 2:** Sequence of primers and probes used in this study.

**Target**	**Name**	**Sequence (5' to 3')**	**Tm (°C)**	**GC** **(%)**	**Amplicon size (bp)**
ASFV	ASFV-F1 ASFV-F2	ACGTAATCCGTGTCCCAACTAA ACATAATCTGTGTCCCAGCTAA	55.9 53.5	45.5 40.9	217
	ASFV-R	CTGCTCATGGTATCAATCTTATCGA	54.4	40.0	
	ASFV-Probe-FAM	CTGGGTTGGTATTCCTCCCGTGGCT	64.4	60.0	
Internal PCR Control (IPC)^*^	IPC-F	CCAGGTTTCCACAGCTGTAGT	57.0	52.5	219
	IPC-R	GGGCTCCATCAAATCTCAGG	55.5	55.0	
	IPC-Probe-HEX	AGCCCTACAACTCCATCCTCACC	60.5	56.5	

* Tubulin alpha a1 gene.

### DNA isolation and optimization of real-time PCR assay

For optimization of the PCR conditions, GT2 synthetic DNA was used as a template. To mimic clinical samples, 30 ng of swine genomic DNA was included in each of the PCR mixture as a background genomic DNA. Swine genomic DNA was purified from pork meat using a DNeasy Blood and Tissue Kit (Qiagen, Germany), according to the protocol provided by the supplier. Concentrations of primers, probe and MgCl_2_ were optimized to achieve maximum amplification efficiency and minimal threshold cycle (Ct). The reaction mixture contained 5 μL of DNA extracts, 5.0 mM of MgCl_2_, 1.6 units of *Taq* DNA polymerase and 8 × 10^4^ copies of a plasmid DNA encoding the IPC target in 50 mM TE, pH8.5 buffer (10 mM Tris and 0.5 mM EDTA, pH 8.5). Concentrations of the primers and probes were as follows: 500 nM of ASFV-F1, 500 nM of ASFV-F2, 500 nM of ASFV-R and 200 nM of ASFV-probe-FAM for ASFV, and 150 nM of IPC-F, 150 nM of IPC-R and 180 nM of IPC-Probe-HEX for IPC.

PCR amplification and real-time detection was performed on Bio-Rad CFX96 real-time PCR Detection System (Biorad, USA). The PCR protocol consists of a hot-start of 95°C for 2 min, followed by 45 cycles of 95°C for 5 s and 56°C for 10 s. Upon completion, amplification graphs and Ct values were recorded. All experiments were performed at least in triplicate.

### Analytical sensitivity

To determine the limit of detection (LoD), serial dilutions the standard template, ASFV DNA GT2 were prepared in TE, pH 9.0 buffer for 5 different concentrations (20, 2, 1, 0.6 and 0.2 copies/μL). For each template concentration, PCR assay was performed in 96-replicates and the positive hit rate with Ct ≤ 40 was determined. The serially diluted standard samples were assayed in triplicate to determine the LoD of the assay by adding 5 μL of each diluted standard sample to the PCR mixture. The resulting concentrations per each PCR mixture were 100, 10, 5, 3 and 1 copies/reaction. In the PCR mixture of 20 μL, 2 μL of porcine genomic DNA (15 ng/μL) was also added to mimic the clinical specimens. The PCR conditions used were described above. The results were analyzed using PROBIT regression analysis.

We also evaluated analytical sensitivity of the assay using serial dilutions of a known viral titer. Viral titer was defined as the amount of virus causing hemadsorption in 50% of infected cultures (HAD_50_/mL). The titer used for serial dilution was 10^5.36^ HAD_50_/mL.

### Cross-reactivity test

Viruses and bacterium used for cross-reactivity test are: Classical swine fever virus (CSFV), *Erysipelothrix rhusiopathiae*, Aujeszky's disease virus (ADV), *Actinobacillus Pleuropneumonia, Salmonella Typhimurium* and *Pasteurella multocida*. DNAs were isolated using QIAamp DNA Mini Kit (Qiagen, Germany) according to the protocol provided by the supplier. Isolated DNA samples were tested to confirm the cross-reactivity of the fast ASFV PCR assay.

### Clinical performance test

Panels of ASFV positive and negative clinical samples of blood and tissues from domestic pigs and wild bores were used for the clinical performance test. The clinical samples consist of fifty positive samples of varying virulence and fifty negative samples that were collected from domestic pigs and wild boars during the year of 2018 to 2019 by National Veterinary Research Institute in Puławy, Poland. The positive samples consisted of 9 blood samples and 41 tissue samples, and the negative samples consisted of 14 blood samples and 36 tissue samples. The tissue samples were from various organs consisting of tonsil, spleen, kidney, lymph node and bone marrow. DNAs from the clinical samples were extracted using QIAamp DNA Mini Kit (Qiagen, Germany) according to the protocol provided by the supplier. Isolated DNA samples were tested to check the clinical performance of the fast ASFV PCR assay.

## Results

### Selection of primers, probes, and optimized parameters for detection of ASFV

The primers and probe for detection of ASFV were designed based on the nucleic acid sequences of all genotypes of the ASFV p72 gene. The gene sequences were acquired from GenBank data base. Several candidate sequences were tried for the primers and probe, and the best results were obtained with ASFV-F1 and ASFV-R1 primers for the genotype group GT2 (data not shown). To cover all 7 groups of ASFV genotypes (24 genotypes), a modified forward primer, ASFV-F2 was included. The sequences of the primers and probe used in this study are listed in Table 2. Equal amounts of ASFV-F1 and ASFV-F2 primers were mixed and used as forward primers. Human tubulin alpha a1 sequence, that is also present in the swine genome, was used as an internal PCR control (IPC), and the primer and probe sequences for detection of IPC are also listed in Table 2.

Annealing temperature and concentrations of primers and probes were optimized for real-time PCR. Optimum annealing temperature was 56°C, and optimum concentrations of primers and probe were 500 nM and 200 nM, respectively, for ASFV and 150 nM and 180 nM, respectively, for IPC. The optimum PCR protocol was as follows: one cycle at 95°C for 2 min, 45 cycles at 95°C for 5 s, then at 56°C for 10 s. Total PCR running time was about 50 min on the Bio-Rad CFX96.

### Limit of detection

**Figure 1:**
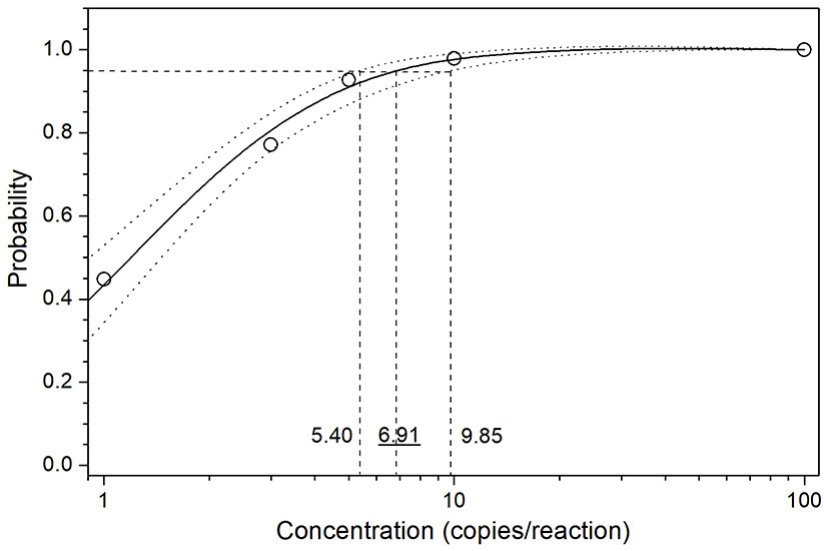
PROBIT regression result for determination of the analytical sensitivity. Serial dilutions of the standard sample template GT2 were used to measure the Ct values and hit rates. The limit of detection (LoD) at 95% hit rate was determined to be 6.91 copies/reaction (5.40~9.85 copies/reaction at 95% confidence interval).

The limit of detection (LoD) of the optimized assay was determined by testing serial dilutions of the standard sample template, ASFV DNA GT2. Ct values and hit rates were measured for 6 different template concentrations (100, 10, 5, 3, 1 and 0 copies/reaction), and the results are listed in Table 3. The test results were analyzed using the PROBIT regression method as shown in Figure 1. The LoD was determined to be 6.91 copies/reaction (5.40 ~ 9.85 copies/reaction at 95% confidence interval).

**Table 3:** Threshold cycle (Ct) values and hit rates of serially diluted samples.

**Concentration** **(copies/reaction)**	**Number of samples**	**Ct**		**Hit rate (%)**
		**Average**	**SD**	
100	96	32.45	1.04	100
10	96	36.34	1.70	97.9
5	96	38.06	2.68	92.7
3	96	38.73	3.22	77.1
1	96	37.69	3.54	44.8
0	48	ND	-	0

The LoD values of the DNA standards for other 6 genotype groups (GT3, GT7, GT8, GT9, GT10 and GT 23) were tested in comparison with the standard sample template, ASFV DNA GT2, and the results are listed in Table 4. GT3, GT8, GT10 and GT23 which include 18 genotypes showed hit rates of > 95% at the concentration of 10 copies/reaction. These values are similar to the value determined for the ASFV DNA GT2 which include 4 genotypes (genotypes I, II, XVII and XVIII). ASFV DNA GT7 and GT9 which include 2 genotypes (genotypes VII and IX) showed a hit rate of 89.6% for 10 copies/reaction, and 95.8 and 100%, respectively, for 20 copies/reaction. The data suggested that LoD of other genotypes is 10~20 copies/reaction or better at ≥95% hit rate.

**Table 4:** Threshold cycle (Ct) values and hit rates of 7 standard genotype groups.

**ASFV group**	**Concentration (copies/reaction)**	**Number of samples**	**Positive**	**Ct**		**Hit rate (%)**
				**Mean**	**SD**	
GT2	10	72	72	35.02	1.28	100.0
GT3	10	48	47	37.86	1.88	97.9
GT7	20	48	46	37.78	2.51	95.8
	10	48	43	38.73	2.85	89.6
GT8	10	48	48	37.67	1.53	100
GT9	20	48	48	35.19	1.43	100
	10	48	43	38.13	4.07	89.6
GT10	10	48	48	36.77	2.05	100
GT23	10	48	48	36.92	1.66	100

Analytical sensitivity of the assay was also determined by testing serial dilutions of a known viral titer (10^5.36^ HAD_50_/mL). The last detectable dilution was 10^4^ as shown in Table 5, corresponding to the analytical sensitivity of 10^1.36^ HAD_50_/mL.

**Table 5:** Analytical sensitivity measured with a known viral titer.

**Virus**	**Genotype**	**Virus titer** **(HAD_50_/mL)**	**Dilution**	**Ct (ASFV)**	**Ct (IPC)**	**Results**
		10^5.36^	1	24.25	26.11	Positive
		10^4.36^	10	27.85	24.80	Positive
ASFV	II	10^3.36^	10^2^	31.77	26.15	Positive
		10^2.36^	10^3^	34.76	25.91	Positive
		10^1.36^	10^4^	39.16	26.20	Positive
		10^0.36^	10^5^	No Ct	26.01	Negative

### Standard curve

**Figure 2:**
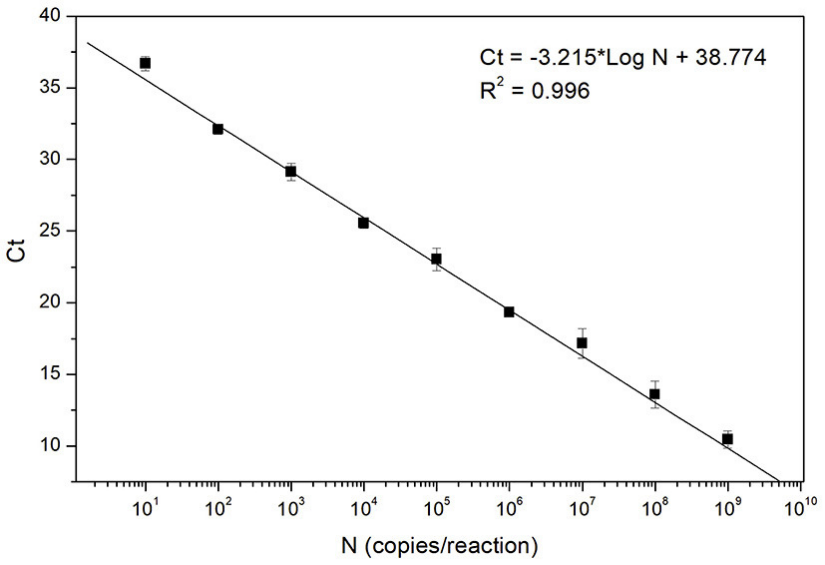
Standard curve of the fast ASFV PCR assay. The curve was generated by analyzing triplicate of 9-fold serial dilutions of the standard sample template, ASFV DNA GT2. Each data point (filled square) corresponds to the mean Ct value of the three replicates at each concentration.

The analytical sensitivity was further studied by testing triplicate of 9-fold serial dilutions of the standard sample template, ASFV DNA GT2. The experiments were performed in the presence of 30 ng of porcine genomic DNA to mimic the clinical test condition. A standard curve was generated by plotting the mean Ct value of the three replicates against the template concentration (10–10^9^ copies/reaction). As shown in Figure 2, a linear regression analysis represents a high coefficient of determination (0.996), demonstrating a linear dynamic range across the 9 orders of magnitude tested, ranging from 10^9^ to the 10 copies/reaction. This standard curve result confirms that the fast ASFV PCR assay developed in this study can be used for quantitative determination of the template concentration. The analytical sensitivity estimated from the standard curve (down to 10 copies/reaction) is also in agreement with the result of the LoD study described above.

### Cross-reactivity of the assay

The cross-reactivity was tested for Classical swine fever virus (CSFV), *Erysipelothrix rhusiopathiae*, Aujeszky's disease virus (ADV), *Actinobacillus Pleuropneumonia, Salmonella Typhimurium* and *Pasteurella multocida*. DNAs were extracted from each microorganism and tested for cross-reactivity. As shown in Table 6, none of the pathogens showed positive results with the assay developed, while positive Ct values were observed for all of the pathogens, confirming no cross-reactivity for the pathogens tested.

**Table 6:** Cross- reactivity of the ASFV assay kit.

**No**.	**Sample**	**Ct**	
		**ASFV**	IPC^*^
1	Classical swine fever virus (CSFV)	No Ct	23.98
2	*Erysipelothrix rhusiopathiae*	No Ct	25.38
3	Aujeszky's disease virus (ADV)	No Ct	24.66
4	*Actinobacillus Pleuropneumonia*	No Ct	24.62
5	*Salmonella Typhimurium*	No Ct	27.83
6	*Pasteurella multocida*	No Ct	24.82

* IPC, internal PCR control.

### Clinical performance test

Clinical performance of the assay was tested with ASFV clinical samples which consist of 50 positive and 50 negative clinical samples of blood and tissues collected from domestic pigs and wild bores during the year of 2018 to 2019. The samples were previously tested and confirmed by National Veterinary Research Institute in Pulawy, Poland. For the test, DNAs were extracted from the clinical samples and used for the PCR assay.

The results of the clinical performance test are listed in Table 7. The results of the fast ASFV PCR assay of this study show 100% agreement with the original diagnosis results: All 50 positive samples were detected to be positive and all 50 negative samples were detected to be negative. Therefore, it is confirmed that both sensitivity and specificity are 100%.

**Table 7:** Clinical sensitivity and specificity.

			**Results of original diagnosis**		**Total**
			**Positive**	**Negative**	
Fast ASFV PCR assay of this study	Positive	Blood	9	0	50
		Tissue	41	0	
	Negative	Blood	0	14	50
		Tissue	0	36	
Total			50	50	100

Clinical sensitivity: 100% (92.9%–100% at 95% confidence interval).

Clinical specificity: 100% (92.9%–100% at 95% confidence interval).

Agreement with the standard real-time PCR method [Fernández-Pinero method using UPL-162 probe (26)] recommended by European Union Reference Laboratory (EURL) in Valdeolmos, Spain and by the International Animal Health Organization (OIE), was also tested. As shown in Table 8, the results of the fast ASFV PCR assay of this study are in 100% agreement for both positive and negative samples with those of the Fernández-Pinero method.

**Table 8:** Agreement with Fernández-Pinero method (UPL-162 probe).

			**Fernadez-Pinero method (UPL-162 probe)**		**Total**
			**Positive**	**Negative**	
Fast ASFV PCR assay of this study	Positive	Blood	9	0	50
		Tissue	41	0	
	Negative	Blood	0	14	50
		Tissue	0	36	
Total			50	50	100

Positive Percent Agreement (PPA): 100% (92.9–100% at 95% confidence interval).

Negative Percent Agreement (NPA): 100% (92.9–100% at 95% confidence interval).

Overall Percent Agreement (OPA): 100% (96.3–100% at 95% confidence interval).

## Discussion

The average time of ASF symptom onset after exposure to ASFV was about 5 to 13 days and death of the infected pigs began to occur at 8–15 days after exposure (11, 22). The time period from the onset of the disease to death of the animal is relatively short and the morbidity and mortality rate is almost 100% (2–4). Moreover, due to the recent increase in the international trade, animal transport and human travel, the risk of transboundary spreading of infectious diseases became significantly higher. Therefore, fast and timely diagnosis and treatment of suspected pigs became more important. In other aspect, infections with different viruses cause similar clinical signs in swine which makes it difficult to diagnosis (17, 30, 31). Therefore, highly specific identification of syndromic pathogens is also required.

Currently, PCR is the most widely used diagnostic techniques for detecting ASF because of its high sensitivity and specificity. Both conventional and real-time PCR assays have been recommended for diagnosis of ASF by OIE. Although these two PCR methods have been used widely, they are either not fast enough or not sufficiently sensitive. In the present study, a highly sensitive, time-saving quantitative real-time PCR technique was developed. The LoD of the assay was 6.91 copies/reaction which is much lower than those of the previously reported values ranging from several 100 and several tens of copies/ reaction (14, 27, 28). The LoD measured with known viral titer was 10^1.36^ HAD_50_/mL that is at least two-times lower than other PCR methods. One study showed a similar LoD of 6 copies/reaction which is comparable with the data presented in this study (29). The LoD value achieved with the assay developed was much lower than those of the conventional PCR Assay (15–17), LAMP assay (18, 19), and RPA assay (22, 23).

When designing primers and probes for molecular diagnostic assays, it is important to select highly conserved regions of viral genome to ensure the assay can detect all known variants or genotypes of the virus. We designed the primers to detect all 24 genotypes of ASFV that are currently identified, and confirmed that all 24 genotypes can be detected with sufficient analytical sensitivity. No cross-reactivity was observed with other swine pathogens and the pathogens of related diseases. It was also demonstrated that the linear dynamic range of this assay is 9-log orders of concentration, sufficient for quantitative determination of ASFV DNA.

The clinical performance was evaluated with 50 positive and 50 negative clinical samples of blood and tissues collected from domestic pigs and wild bores. The test results obtained with clinical samples revealed that the assay has 100% sensitivity and 100% specificity, and also 100% PPA and 100% NPA when compared with the standard Fernández-Pinero method. The whole PCR process to results can be completed in about 50 min which is at least about two-times shorter than the 90~120 min running time of other real-time PCR assays (14, 25, 28, 29).

In summary, a real-time PCR assay has been developed that is faster, more sensitive than currently available PCR methods and can detect all 24 genotypes of ASF. The results of the analytical and clinical performance tests revealed that the assay is much faster and more sensitive than other PCR methods and represents high specificity with no cross-reactivity. The assay developed in this study can be a useful molecular diagnostic tool for the prompt control and prevention of ASF.

## Data availability statement

The original contributions presented in the study are included in the article/supplementary materials, further inquiries can be directed to the corresponding author.

## Ethics statement

The animal study was reviewed and approved by National Veterinary Research Institute, Al. Partyzantów 57, 24-100 Puławy, Poland.

## Author contributions

HJH contributed to the idea conception. YSC and KS performed the analytical data collection and analysis. MF performed the clinical data collection and analysis. JHK and HJH contributed to design of the study and analysis of the data. JHK supervised the experiments. HJH and JHK prepared the manuscript. All authors contributed to the article and approved the submitted version.

## Funding

This work was supported by Korea Evaluation Institute of Industrial Technology and funded by the Ministry of Trade, Industry and Energy, Korea (Grant 10080151).

## Conflict of interest

Authors HJH, YSC, and KS were employed by Ahram Biosystems, Inc. The remaining authors declare that the research was conducted in the absence of any commercial or financial relationships that could be construed as a potential conflict of interest.

## Publisher's note

All claims expressed in this article are solely those of the authors and do not necessarily represent those of their affiliated organizations, or those of the publisher, the editors and the reviewers. Any product that may be evaluated in this article, or claim that may be made by its manufacturer, is not guaranteed or endorsed by the publisher.
